# Reverse Design of Pixel-Type Micro-Polarizer Arrays to Improve Polarization Image Contrast

**DOI:** 10.3390/mi15101251

**Published:** 2024-10-12

**Authors:** Yonggui Shi, Zhihai Lin, Tianran Wang, Chaokai Huang, Hui Chen, Jianxiong Chen, Yu Xie

**Affiliations:** 1 School of Intelligent Manufacturing, Xiamen City University, Xiamen 361008, China; shiyonggui@xmcu.edu.cn; 2 School of Mechanical Engineering and Automation, Fuzhou University, Fuzhou 350108, China

**Keywords:** MPA, reverse design, particle swarm optimization, DOLP image contrast, optimizing optical performance

## Abstract

Micro-polarizer array (MPA) is the core optical component of the Division of Focal-Plane (DoFP) imaging system, and its design is very important to the system’s performance. Traditional design methods rely on theoretical analysis and simulation, which is complicated and requires designers to have profound theoretical foundations. In order to simplify the design process and improve efficiency, this paper proposes a 2 × 2 MPA reverse-design strategy based on particle swarm optimization (PSO). This strategy uses intelligent algorithms to automatically explore the design space in order to discover MPA structures with optimal optical properties. In addition, the all-pass filter is introduced to the MPA superpixel unit in the design, which effectively reduces the crosstalk and frequency aliasing between pixels. In this study, two MPA models were designed: a traditional MPA and an MPA with an all-pass filter. The Degree of Linear Polarization (DOLP) image contrast is used as the evaluation standard and compared with the traditional MPA; the results show that the contrast of the newly designed traditional MPA image is increased by 21%, and the MPA image with the all-pass filter is significantly increased by 82%. Therefore, the reverse-design method proposed in this paper not only simplifies the design process but also can design an MPA with enhanced optical performance, which has obvious advantages over the traditional method.

## 1. Introduction

As a characteristic of light, polarization imaging can provide rich information about target features. Currently, polarization imaging technology is widely utilized in military covert target reconnaissance [1], haze elimination [2], the enhancement of unknown target images [3], astronomical observation [4], medical image enhancement [5], natural resource detection [6], and various other fields. The existing polarization imaging systems can be categorized into four types: Division of Time Polarimeter (DoTP), Division of Aperture Polarimeter (DoAP), Division of Amplitude Polarimeter (DoAmP), and Division of Focal-Plane Polarimeter (DoFP) [7]. The DoTP polarimeter requires a rotating polarizer, making it suitable only for static scenes. The DoAmP polarimeter utilizes multiple focal plane arrays (FPA) to obtain measurement data simultaneously, but its optical system is large, complex, and expensive. The DoAP polarimeter can simultaneously capture polarization information from the same target in different directions on a detector focal plane, but it suffers from expensive optical components and spatial resolution loss. In comparison to the other three polarization imaging systems, the DoFP polarimeter integrates a micropolarizer array (MPA) and a detector focal-plane array (FPA), offering outstanding advantages such as snapshot imaging, a compact structure, low power consumption, high transmission efficiency, and a high extinction ratio [8]. However, loss of spatial resolution and the error in the instantaneous field of view are inherent problems in DoFP polarimetry [9]. To address these issues, various interpolation methods such as bilinear interpolation, bicubic interpolation, and gradient-based interpolation have been employed to correct polarization images, thereby enhancing resolution [10,11,12]. Simultaneously, the study of high-performance MPA as the core optical component in DoFP polarimetry has also emerged as a critical avenue for improving the imaging quality of DoFP.

To enhance the performance of the MPA, extensive research has been conducted on its design. Dmitry V [13] improved device performance by adjusting the structural parameters of the metal grating. Zhao et al. [14] introduced two new superpixel units for the “quasi-Bayer” MPA based on the “Bayer” coding mode. Experimental results demonstrate a significant improvement in the imaging quality of the new MPA. AS Alenin [15] designed the superpixel unit of the MPA using a specific coding method combined with Fourier frequency theory and numerical simulation. The experimental results indicate good polarized image quality for the designed 2 × 3, 2 × 4, and 2 × 2 × 2 MPAs.

Conventional design methodologies exhibit a pronounced dependence on the experiential acumen of designers and foundational optical principles. These methods predominantly involve the iterative adjustment of structural parameters within devices, aiming to attain specific performance criteria such as those pertaining to polarizers [16], terahertz (THz) filters [17], and demultiplexers [18]. The contemporary imperative underscores the pursuit of miniaturized, integrated, and high-performance MPA.

In contrast, the reverse-design method (RDM) represents a distinctive and innovative paradigm. This approach entails the consideration of target performance as a benchmark for evaluation, employing intelligent algorithms to iteratively optimize the initial structural configuration. The ultimate objective is the identification of a device that satisfies the specified performance criteria. Diverging from conventional methodologies, this novel approach is characterized by its exhaustive exploration of the entire design space, enabling the realization of devices endowed with enhanced capabilities, intricate functionalities, and superior performance [19,20].

Recent advancements in optical device design showcase the widespread adoption of RDM. Examples include the design of pixel-type terahertz band-pass filters through the discrete binary particle swarm optimization (BPSO) algorithm [16], a photonic crystal fiber-based polarization filter proposed via artificial neural networks and intelligent optimization algorithms [21], and a microstructure of deep ultraviolet light-emitting diodes (DUV-LEDs) developed utilizing the particle swarm optimization algorithm [22], among others. Notwithstanding the success achieved in various optical applications, the application of RDM in the realm of MPA design remains comparatively limited.

In this paper, we propose an RDM for an MPA based on the particle swarm optimization (PSO) algorithm. This method takes into account the structural parameters of the metal grating and the arrangement of superpixel unit coding combinations, thereby improving the performance of the MPA through these two factors. This automated design method can explore all potential design configurations within a specific parameter range, thereby enhancing the likelihood of discovering superior performance configurations. Furthermore, this method enables online MPA design, allowing for performance analysis through simulation. As a result, this approach can significantly reduce design costs.

The structure of this paper is as follows: Section 2 introduces the key parameters that define MPA and outlines its design principles. Section 3 details our proposed PSO-based reverse-design method for MPA. In Section 4, we present experimental results demonstrating the advantages of our design approach. Finally, Section 5 provides the concluding remarks.

## 2. Key Parameters of MPA

The MPA is comprised of periodic superpixel units, each consisting of metal gratings oriented in various directions. The imaging of the MPA is a complex process that can be influenced by various factors, including the structural parameters of the metal grating and the coding of superpixel units. Hence, the design of the metal grating and the coding of superpixel units are of utmost importance.

### 2.1. Metal Grating Structure

**Figure 1:**
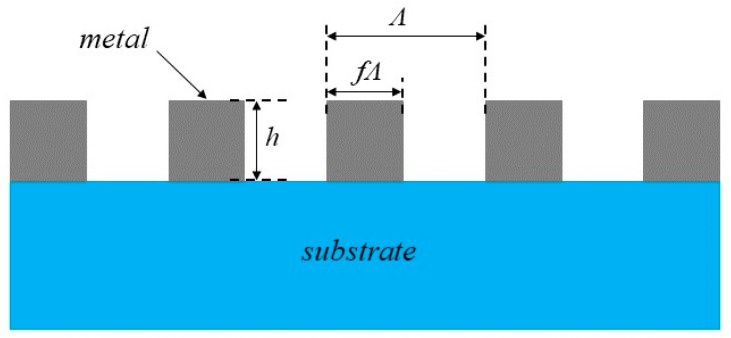
Schematic diagram of the metal grating structure.

Various factors must be considered when designing the metal grating [23] to achieve the desired optical properties and fabrication feasibility. As depicted in Figure 1, h represents the height of the gratings, and Λ denotes the period of the gratings, which influence the spatial and spectral resolution of the gratings. The grating height should be selected considering manufacturing feasibility and performance requirements. A larger grating height will increase manufacturing difficulty, while a smaller grating height will reduce the grating’s performance. The *f* is the duty cycle. It was found in [24] that when *f* is about 0.5, the grating has good performance. Hence, the *f* is set to 0.5. Aluminum was selected as the material due to its alignment with the specified criteria for grating polarization performance. Then, through software simulation, the transmittance and extinction ratio of the grating can be obtained.

### 2.2. Superpixel Unit Coding

**Figure 2:**
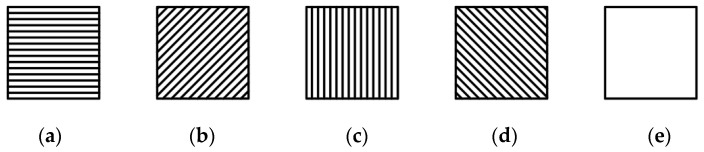
Grating units at different angles in the MPA: (**a**) 0° grating unit, (**b**) 45° grating unit, (**c**) 90° grating unit, (**d**) 135° grating unit, and (**e**) full-pass filter, which passes all states of light equally.

**Figure 3:**
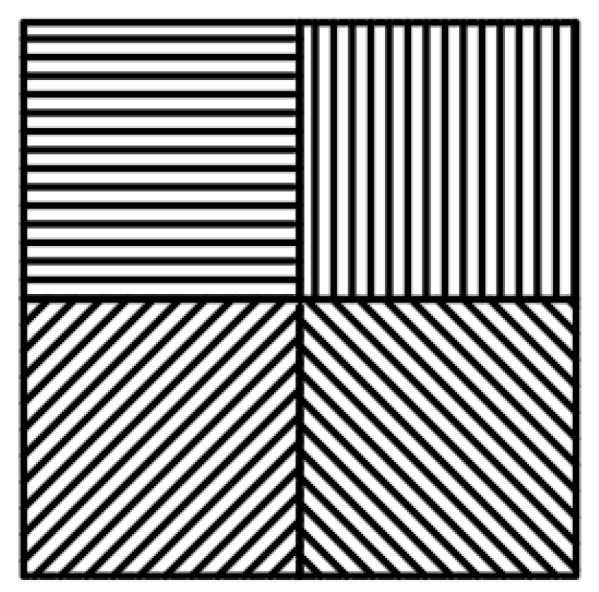
Conventional 2 × 2 superpixel units.

Traditional MPA consists of periodic superpixel units, each of which is composed of metal grating units with orientation angles of θ = {0,45,90,135}° [25]. Figure 2 illustrates the metal grating elements at different angles in the MPA. These grating units are organized and combined into 2 × 2 superpixel units, as shown in Figure 3. These superpixel units are then periodically arranged to create a traditional MPA. The traditional MPA is widely used in infrared polarization-imaging systems. However, it faces a common issue: a high pixel crosstalk rate that negatively impacts image quality. This problem is primarily caused by the large polarization components on the MPA, the dense distribution of gratings, the minimal spacing between adjacent gratings, and the differing polarization intensity information they provide. These factors lead to crosstalk between pixel units and frequency aliasing, which results in a loss of detailed information and a decrease in image contrast [26].

To minimize the impact of crosstalk on the MPA performance, the design of the coding combination for superpixel units in the MPA should adhere to the following principles [27]:

(1) Utilize a reduced number of grating units. For instance, each superpixel unit should incorporate grating units with only two angles. This approach lowers the overall polarization component of the MPA, thereby diminishing the occurrence of frequency aliasing and mitigating pixel crosstalk.

(2) Incorporate the full-pass filter illustrated in Figure 2e. The addition of the full-pass filter increases the spacing between the different grating units within the superpixel unit, effectively reducing the interference caused by frequency aliasing and minimizing pixel crosstalk.

Consequently, this paper employs the four angles of the grating units and the full-pass filter (Figure 2e) for the coding of superpixel units within the MPA.

## 3. Reverse Design of MPA

The conventional forward design methodology typically relies on specific analytical theories. For instance [28], the performance of the metal grating is analyzed using effective medium theory and the finite-difference time-domain (FDTD) method. It is found that performance of the metal grating can be optimized by altering parameters such as the grating material, grating period, and grating height. Different grating parameters are adopted, followed by simulation analysis using FDTD software (2020 R2.4) until the grating structure with the best optical performance is achieved. This method, however, is not only time-consuming and labor-intensive but also demands a considerable depth of experience in theoretical analysis. In the context of nonlinear device optimization, the traditional approach involves optimizing several interdependent characteristic parameters simultaneously, relying heavily on individual expertise. In contrast, RDM treats the functional parameters of the device as evaluation indices and introduces intelligent algorithms to identify optimal structures. By exploring the entire design space using intelligent algorithms, RDM facilitates the discovery of non-periodic topological device structures, enabling the realization of complex functions and enhanced performance that were previously unattainable.

Among the existing optimization algorithms, the genetic algorithm, the simulated annealing algorithm, and the particle swarm optimization algorithm are widely used in process parameter optimization problems [29]. Heuristic optimization algorithms such as simulated annealing and genetic algorithms are popular in applications, especially in discrete optimization problems. Unfortunately, the practical efficiency of heuristic optimization algorithms has large defects [30]. The PSO algorithm is simple to implement, has fast convergence speed and a strong global search ability, and performs well in optimizing nonlinear and multimodal functions [31].

Therefore, this paper proposes an MPA RDM based on the PSO algorithm, comprising three main parts. Section 3.3 is the core of the reverse-design method for MPAs, while Section 3.1 and Section 3.2 provide the essential groundwork for the entire reverse-design process.

### 3.1. Determine the Metal Grating Design Function

The critical parameters of metal gratings include grating period, grating height, and superpixel unit coding, which affect the performance of metal gratings. The simulation software is adopted to analyze the influence of grating height x and grating period y on the transmittance z of polarization gratings. Then, the relationship between grating height, grating period, and grating transmittance is obtained by fitting the data. The derived function is referred to as the “metal grating design function”.

### 3.2. Acquisition of DOLP Images and Its Contrast Value

Degree of Linear Polarization (DOLP) image acquisition methods include time-sharing polarization imaging and simultaneous polarization imaging [32]. The current approach for simultaneous polarization imaging involves integrating the MPA studied in this paper with the detector to capture various polarization information of the target simultaneously. However, if the aim is to assess the performance of the different coding combinations of the MPA, it would significantly increase the experimental cost. Therefore, this paper opts for the time-sharing polarization-imaging method. Following the principles of time-sharing polarization imaging, polarization images of the same target with different polarization angles are collected at various time points. Using OpenCV, the obtained polarization images undergo processing, such as pixel extraction and reorganization of superpixel units, ultimately resulting in simultaneous polarization images of the MPA with different superpixel units.

**Figure 4:**
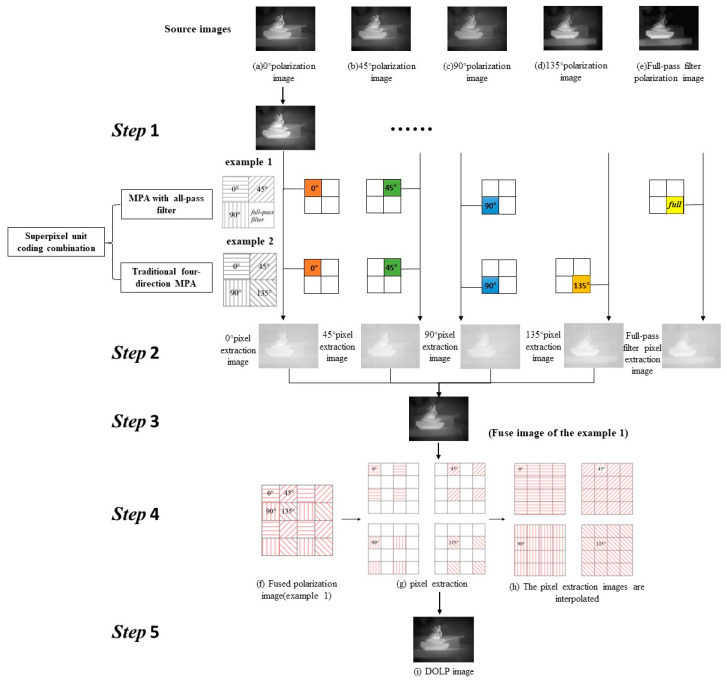
Flow chart of the DOLP image acquisition for the MPA.

To obtain the DOLP image of the MPA with a specific transmittance and coding combination, five steps are needed (Figure 4):

S1: Perform equivalent transformation on the source images according to the input transmittance (75%). Figure 4a–e shows the source images acquired through the time-sharing polarization-imaging system. The source image was equivalently transformed according to the input transmittance. The principle of the equivalent transformation is represented as:

(1) $$\frac{z_{0}}{z_{in}}=\frac{I_{0}}{I_{in}}.$$

where $z_{0}$ is the transmittance of the infrared polarizer (75%), $z_{in}$ is the transmittance of input, $I_{0}$ is the light intensity of the polarization image corresponding to a transmittance of 75%, and $I_{\mathrm{in}}$ is the light intensity of the polarization image corresponding to the input transmittance *Z_in_*.

S2: Perform the pixel extraction according to the coding combination. As shown in Figure 4, the pixels were extracted from the equivalent transformed polarization image obtained in the previous step.

This paper mainly optimized the design of the traditional MPA and the MPA with an all-pass filter. Therefore, the input superpixel unit coding combination is mainly divided into two groups. The first group is the superpixel unit coding combination of the traditional MPA, involving the combination of four grating units with different angles, as shown in Figure 2a–d. There are a total of 24 coding combinations in this category. The second group is comprised of coding involving full-pass filters, where any three grating units from (a) to (d) in Figure 2 were arranged and combined with full-pass filters. This category includes a total of 96 coding combinations. It should be noted that the traditional four-direction MPA was designed separately from the MPA containing the full-pass filter. The traditional four-direction MPA and the MPA with the full-pass filter in step 2 of Figure 4 are only used as examples of pixel extraction and were not performed simultaneously.

S3: Fuse the pixel extraction images. The pixel extraction images from the four angles obtained in the previous step were combined into a new polarization image through image processing.

S4: Split and interpolate the fused polarization image. Extract the pixel values with the same polarization angle from the polarization image obtained in S3. Assign these pixel values to the corresponding positions on a prepared blank image of the same size. This results in four new images containing unknown pixels, as illustrated in Figure 4g. Each of the four new images contains only 25% of the pixel values from the original image, with the remaining 75% being unknown. To obtain all pixel values, we utilized the bilinear interpolation algorithm to calculate the unknown pixel values based on the neighboring four known pixel values [33]. Figure 4f–h depicts the splitting and interpolating of the fused polarization image.

S5: Obtain the DOLP image and its contrast value. The interpolated images in Figure 4h were derived using the Stokes vector method to obtain the DOLP image [34]. The contrast value of DOLP image was then calculated.

### 3.3. Particle Swarm Optimization Algorithm

The particle swarm optimization (PSO) algorithm [31] is a widely used swarm intelligence algorithm that draws inspiration from the study of bird foraging behavior. When a flock of birds searches for food, the simplest and most effective strategy is to explore the area around the bird that is closest to the food within the flock.

In PSO, each potential optimal solution to the optimization problem is treated as a particle, and these particles move within the solution space to seek the optimal solution. The velocity and position of the particle constitute two crucial variables in the algorithm. The update of velocity and position governs the search direction and region of the particle within the solution space, making it a pivotal process in the particle swarm optimization algorithm. During the search process, each particle keeps a record of the best position it has reached, known as its individual historical optimal solution, while the swarm collectively updates its best position, referred to as the swarm optimal solution. In each iteration, every particle adjusts its speed and position based on its current speed and position, taking into account the information from its individual historical optimal solution and the swarm’s optimal solution. The velocity and position update rules for particles are depicted in Equations (2) and (3):

(2) $$v_{i,j}^{t+1}=w\cdot v_{i,j}^{t}+c_{1}\cdot r_{1}\cdot \left({\mathrm{pbest}}_{t,j}-x_{i,j}^{t}\right)+c_{2}\cdot r_{2}\cdot \left({\mathrm{gbest}}_{t,j}-x_{i,j}^{t}\right)$$

(3) $$x_{i,j}^{t+1}=x_{i,j}^{t}+v_{i,j}^{t+1}$$

where $v_{i,j}^{t+1}$ represents the velocity of particle i in the (*t +* 1)th iterations, $x_{i,j}^{t}$ denotes the position of particle i in (*t +* 1)th iterations, w is the inertia weight, $c_{1}$ and $c_{2}$ are the acceleration constants, $r_{1}$ and $r_{2}$ are random numbers between 0 and 1, ${\mathrm{pbest}}_{t,j}$ is the individual historical optimal solution of particle *i* in dimension *j*, and ${\mathrm{gbest}}_{t,j}$ is the group optimal solution of particle swarm in dimension *j*.

PSO employs a fitness function to assess the fitness of each particle, which guides its position and velocity adjustments within the search space [35]. In PSO, the fitness function is typically defined as the objective to be optimized. Simultaneously, the fitness function holds significant importance in PSO as it directly influences the algorithm’s convergence speed and the quality of the final result.

In the field of image processing, contrast [36] in grayscale images is a crucial visual feature, one that can directly impact the image’s quality and readability. In general, the higher the contrast in an image, the clearer its details, the more pronounced its edges, and the easier it is to analyze and process the image. Therefore, this paper adopted the contrast of the DOLP image as the fitness function. The image contrast is presented as:

(4) $$C=\frac{\sqrt{\frac{1}{N}\sum_{x,y}{\left[I(x,y)-\bar{I}\right]}^{2}}}{\bar{I}}$$

where *N* is the total number of pixels in the image, *I (x, y)* is the intensity value of the pixel, and *I* represents the average gray value of the target image.

Therefore, the main purpose of this section was to obtain the MPA with the best imaging performance through PSO optimization algorithm. The grating height, grating period, and different superpixel unit codes were used as input parameters, and the contrast calculation formula of the DOLP image of the MPA was used as the fitness function for the optimization. Among them, the imaging performance was measured by the DOLP image contrast of the MPA, so the calculation formula of the DOLP image contrast was used as the fitness function of the PSO optimization algorithm.

**Figure 5:**
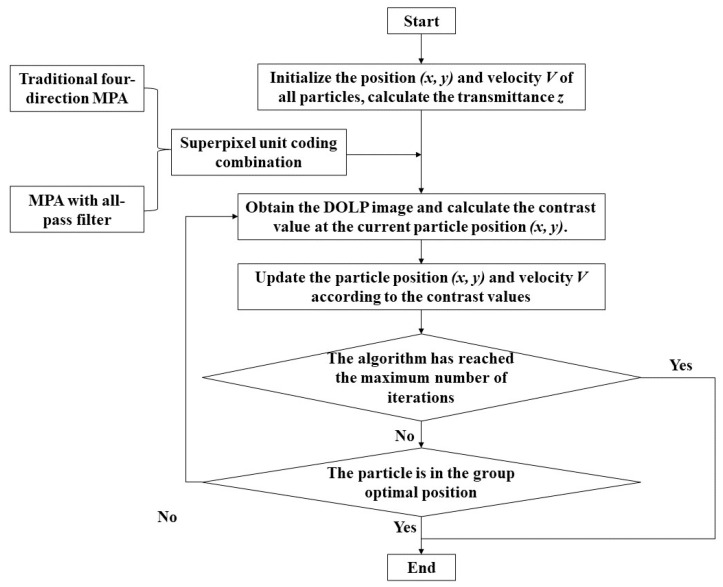
Flow chart of the particle swarm optimization algorithm.

The flow chart of the particle swarm optimization algorithm is shown in Figure 5. The primary steps for designing the coding combination of superpixel units in the MPA using the particle swarm optimization algorithm are as follows:

Step 1: Initialize the position (x, y) and velocity V of all particles in the population. The position of each particle in the search space can be represented by (x, y), where x represents the grating height, and y represents the grating period. Then, the value of transmittance *Z_in_* is calculated by the coordinates (x, y) and the grating design function. The particle’s position should be constrained within the range x = (0.1, 1.1) and y = (0.4, 5.0).

Step 2: Calculate the fitness value at the current particle position (x, y). DOLP images of the MPA are acquired for the given grating period x, grating height y and various coding combinations. The contrast values of the DOLP images are computed, and these calculated contrast values are then provided to the particle swarm optimization algorithm as the fitness values. Finally, the individual historical optimal solution pbest and the group optimal solution gbest are updated in real time by comparing the fitness values.

Step 3: Update the particle position (x, y) and velocity V according to Equations (2) and (3).

Step 4: Determine whether the particle is in the group’s optimal position and whether the algorithm has reached the maximum number of iterations. If all the conditions are satisfied, the algorithm stops running; if not, it returns to Step 2.

## 4. Results and Discussion

### 4.1. Simulation Experiment

**Figure 6:**
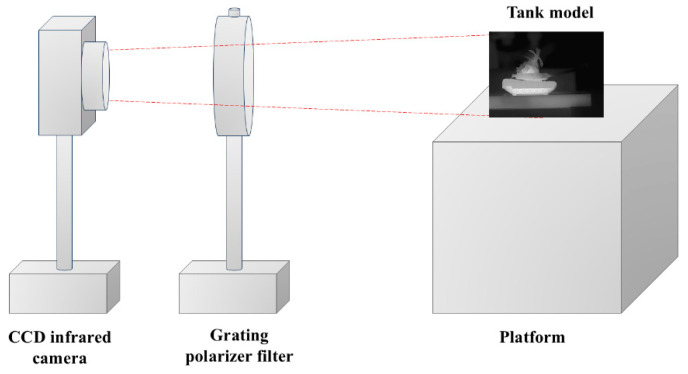
Time-sharing, long-wave infrared polarization-imaging system.

In this paper, a time-sharing, long-wave infrared polarization-imaging system was designed to collect configuration images with different polarization angles, as depicted in Figure 6. In the imaging system, the detector was a CCD infrared camera (384 × 288 pixels; XI370-F190M; Chengdu Jinglin Company; Chengdu, China). The detector’s spectral response band covered 8~14 μm, and it employed a USB data interface to fulfill the demands of real-time image acquisition and data transmission.

The infrared polarizer filter utilized a metal grating polarizer produced by Edmund Company (Barrington, NJ, USA) with BaF2 as the substrate. The substrate featured a uniform distribution of 1200 lines/mm metal grating, corresponding to a light-wave frequency band of 1.5–12 μm. The effective transmittance of the metal grating was approximately 75%, with an extinction ratio of 300:1. The time-sharing imaging system was employed to capture polarization images of the tank model from four different angles, as illustrated in Figure 2a–e. Among them, Figure 2e displays the image of the full-pass filter in the MPA directly collected by the CCD infrared camera. The CCD infrared camera was paired with the infrared polarizer to record the respective polarization images after rotating the polarizer to 0°, 45°, 90°, and 135°, as shown in Figure 2a–d.

After collecting the polarization images, the next step was to calculate the grating design function. Simulation software was adopted for simulation calculations. During the simulation, the incident wavelength was set to 10.6 µm, with the grating height ranging from 0.1 µm to 1.1 µm in steps of 0.1 µm. The grating period ranged from 0.4 µm to 5.0 µm, also in steps of 0.1 µm. This resulted in 517 combinations of grating height and grating period. Aluminum was selected as the grating material. The transmittance for each of these 517 combinations was then simulated.

**Figure 7:**
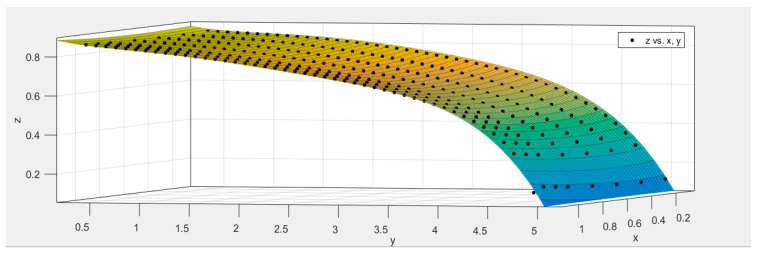
The fitted surface of the design function of the metal aluminum grating.

Subsequently, the data from these 517 groups of simulations were divided into two sets: the model training group (450 groups) and the model testing group (67 groups). Each group’s data included three parameters: grating height x, grating period y, and grating transmittance z. These three parameters were then fitted. This process yielded a fitting surface and a polynomial related to the three parameters. In this paper, polynomial functions were chosen for fitting, with the highest order of fitting set to 5. Equation (5) represents the polynomial formula after fitting, which served as the metal grating design function. Table 1 contains the polynomial coefficients, and the fitted function is displayed in Figure 7, where the change in color represents the change in height (that is, the value of the z-axis).

(5) $$\begin{array}{l} f\left(x,y\right)=p_{00}+p_{10}\times x+p_{01}\times y+p_{20}\times x^{2}+p_{11}\times x\times y+p_{02}\times y^{2}+p_{30}\times x^{3}+p_{21}\times x^{2}\times y\\ +p_{12}x\times y^{2}+p_{03}\times y^{3}+p_{40}\times x^{4}+p_{31}\times x^{3}\times y+p_{22}\times x^{2}\times y^{2}+p_{13}\times x\times y^{3}+p_{04}\times y^{4}\\ +p_{50}\times x^{5}+p_{41}\times x^{4}\times y^{1}+p_{32}\times x^{3}\times y^{2}+p_{23}\times x^{2}\times y^{3}+p_{14}\times x\times y^{4}+p_{05}\times y^{5} \end{array}$$

Table 2 presents the evaluation value of the fitted surface, which indicates the fitting accuracy. As shown in Table 2, the SSE and RMSE for the model training group are both very small, at 0.0475 and 0.0125, respectively. The R-square and Adjusted R-square values are both close to 1, measuring 0.9961 and 0.9959, respectively. Thus, it is evident that the fitting results are accurate.

Then, the mean square error [37] between the predicted values and the true values is calculated as:

(6) $$MSE=\frac{1}{n}{\sum_{i=1}^{n}\left(Y_{i}-\bar{Y_{i}}\right)}^{2}$$

*MSE* equals 0.0115, signifying that the error between the fitting function and the original data is minimal, indicating an excellent fitting accuracy of the metal grating design function. Then, the PSO algorithm was executed to optimize both the traditional MPA and the MPA with a full-pass filter.

### 4.2. Results Analysis

**Figure 8:**
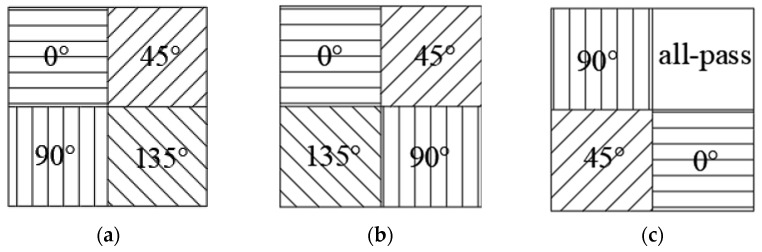
The superpixel unit of the MPA: (**a**) traditional superpixel unit, (**b**) optimized traditional superpixel unit, and (**c**) optimized superpixel unit with an all-pass filter.

**Figure 9:**
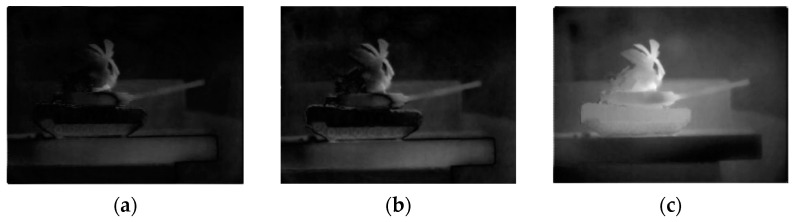
DOLP image of (**a**) a traditional MPA (CV = 69.0041), (**b**) an optimized traditional MPA (CV = 83.442787), and (**c**) an optimized MPA with an all-pass filter (CV = 125.6748).

The PSO algorithm was employed to optimize the design of both the traditional MPA and the MPA with a full-pass filter. Through iterative calculations, it was found that for the traditional MPA, the optimal parameters are a grating height of 0.33 µm; a grating period of 1.72 µm; a grating transmittance of 0.7985; and a coding sequence of 0°, 45°, 135°, and 90° (Figure 8b). Under these conditions, the contrast value (CV) of the DOLP image of the MPA reaches a maximum value of 83.4428 (Figure 9b). For the MPA with a full-pass filter, the optimal parameters are a grating height of 0.38 µm; a grating period of 1.93 µm; a grating transmittance of 0.7871; and a coding sequence of 45°, 0°, full-pass filter, and 90° (Figure 8c). The contrast value (CV) of the DOLP image of the MPA reaches a maximum value of 125.6748 (Figure 9c).

To verify the imaging performance of the designed MPA, a comparative test was conducted using the MPA model designed by Wu Z [38]. The parameters for this model are a grating height of 0.14 µm; a grating period of 0.4 µm; a grating transmittance of 0.8147; and a coding sequence of 0°, 45°, 90°, and 135°. The superpixel unit of this MPA model is shown in Figure 8a, and its DOLP image is shown in Figure 9a. The contrast value of the DOLP image of this model is 69.0041.

Comparing this MPA model with the optimized traditional MPA and the all-pass filter MPA, the contrast value of the polarization image is greatly improved. The contrast value of the DOLP image of the optimized traditional MPA increased by 21%. After the all-pass filter was added to the MPA design, the contrast value of the DOLP image of the MPA increased by nearly 82%.

Additionally, by observing the images in Figure 9a–c, it is evident that the image quality of the DOLP images of the optimized MPA was enhanced, offering richer details and clearer target edges. These improvements facilitate more accurate target identification. This demonstrates that the reverse-design method for the MPA based on the PSO algorithm proposed in this paper is highly effective, significantly enhancing the imaging performance of the MPA designed using this method.

## 5. Conclusions

This paper adopts image contrast as the optimization target and utilizes the coding of the superpixel unit and the metal grating structure as the design parameters. The MPA with the best imaging performance is then realized using a reverse-design method based on the PSO algorithm. The feasibility of this method is confirmed through simulation experiments. This automated design approach explores all possible design schemes within a specific area, increasing the likelihood of discovering superior performance configurations. This method aims to intelligently design an MPA through a reverse-design process, avoiding the need for the extensive theoretical groundwork and tedious parameter optimization inherent in forward-design methods. The results demonstrate a significant improvement in the design efficiency and quality of the MPA. Specifically, the contrast of the newly designed traditional MPA image increased by 21%, while the MPA image using an all-pass filter saw a substantial contrast increase of 82%. Hence, this approach is suitable for the intelligent, miniaturized, and high-performance design of an MPA.

## Figures and Tables

**Table 1:** Fitting polynomial function coefficients of the metal gratings.

Coefficients	Coefficients Value	Confidence Interval
P00	0.9132	(0.7855, 1.0410)
P10	−0.0876	(−0.9639, 0.7887)
P01	−0.1667	(−0.3867, 0.0534))
P20	0.0738	(−3.0840, 3.2310)
P11	0.1075	(−0.3962, 0.6112)
P02	0.1719	(0.0044, 0.3394)
P30	−0.0510	(−5.5780, 5.4680)
P21	−0.0322	(−0.8334, 0.7690)
P12	−0.0987	(−0.2796, 0.0822)
P03	−0.0943	(−0.1565, −0.0321)
P40	0.0809	(−4.5250, 4.6870)
P31	−0.0522	(−0.7325, 0.6281)
P22	0.0522	(−0.0863, 0.1907)
P13	0.0201	(−0.0135, 0.0537)
P04	0.0226	(0.0114, 0.0337)
P50	−0.0299	(−1.5010, 1.4420)
P41	0.0123	(−0.2344, 0.2591)
P32	0.0025	(−0.0493, 0.0542)
P23	−0.0077	(−0.0192, 0.0039)
P14	−0.0010	(−0.0036,0.0017)
P05	−0.0021	(−0.0029, −0.0013)

**Table 2:** Evaluation value of the fitted surface.

Evaluation Index	Evaluation Value
Sum of Squared Errors (SSE)	0.0475
Coefficient of determination (R-square)	0.9961
Adjusted coefficient of determination (Adjusted R-square)	0.9959
Root Mean Squared Error (RMSE)	0.0125

## Data Availability

The data presented in this study are available on request from the corresponding author.
